# In Silico Design and Characterization of a Multi-Epitope Vaccine Candidate Against *Mycoplasma pneumoniae* Using a Reverse Vaccinology Strategy

**DOI:** 10.3390/microorganisms14030567

**Published:** 2026-03-02

**Authors:** Lingling Chen, Yang Li, Wanying Gao, Jiaqi Nie, Xiao Jiang, Henan Cao, Shulei Jia

**Affiliations:** 1 School of Basic Medical Sciences, Tianjin Medical University, Tianjin 300070, China; 2 Institute of EcoHealth, School of Public Health, Cheeloo College of Medicine, Shandong University, Jinan 250012, China

**Keywords:** *Mycoplasma pneumoniae* (Mp), immunoinformatics, multi-epitope, reverse vaccinology, molecular docking

## Abstract

*Mycoplasma pneumoniae* (*M. pneumoniae*) is the crucial factor of global acquired respiratory infections. Currently, there are no specific disease modification treatments or vaccines available, and the vaccine development for this pathogen lags behind due to the complexity and variability of its antigens. A novel vaccine with broad-spectrum characteristics is essential to provide comprehensive protection against continuously evolving wild-type strains. Here, a broad-spectrum muti-epitope vaccine against *M. pneumoniae* had been designed through immunoinformatics methods. To ensure its broad-spectrum, we generated consistent sequences from all the antigen proteins of different strains, and then identified potential T cell epitopes. The multi-epitope vaccine (MEV) of *M. pneumoniae* incorporated 16 CTLs and 7 HTLs from the HMW1–3 and p1 adhesin proteins, which comprised 458 amino acids with adjuvant. The vaccine evaluation showed that the MEV had ideal physicochemical properties, high antigenicity, high immunogenicity, and was non-toxic. Furthermore, there was a strong and stable binding interaction between this vaccine and the toll-like receptors, which could be supported by the normal mode analysis. Finally, codon optimization resulted in the optimal GC content and higher CAI value. The vaccine candidate is expected to induce strong cellular immune responses and may provide protective immunity against the pathogen. We provided a novel in silico vaccine design strategy for vaccine design, which could provide a technical framework for the development of vaccines against other pathogens.

## 1. Introduction

*Mycoplasma pneumoniae* (Mp) is the predominant pathogen responsible for community-acquired pneumonia in humans; its widespread antibiotic resistance therefore renders vaccination the best method to prevent its spread. It is the microorganism that lies between viruses and bacteria, often co-infecting with other viruses [1,2], causing illnesses from mild upper respiratory infections to severe atypical pneumonia, primarily affecting children and young adults. Typically, this disease presents with symptoms related to respiratory and lung infections, and in hospital settings, the mortality rate has been noted as 3% [1,2,3,4]. Up until now, a large number of reports have been published on the relationships between *M. pneumoniae* and the networks of community-acquired infections [5,6,7]. Although the investigation report offers a wealth of data, managing and treating *M. pneumoniae* infection effectively continues to be difficult. The limited biosynthetic capability and slow replication speed of *M. pneumoniae* are due to its extremely small genome [8,9,10]. *M. pneumoniae* transmits via respiratory droplets that interact with mucosal surfaces, often leading to localized inflammation and related pathological effects [11]. At the initial stage of *M. pneumoniae* infection, the organism attaches to the columnar epithelium of ciliated cells, shielding itself from local cytotoxic effects and mucociliary clearance [6]. A specialized organelle facilitates cell adhesion to sialic acid glycoproteins and sulfate glycolipids, featuring a central core of dense filaments and a pointed structure of adhesins and accessory proteins [12,13]. The key proteins such as the p1 adhesin, p30 adhesin, and p65 adhesin are involved in binding to receptors. The high molecular weight proteins HMW-1, HMW-2, and HMW-3 interact with the adhesins p1, p30 and p65 to aid in cell fusion [14,15]. These proteins could be targeted by drugs for effective treatment and vaccine development to prevent *M. pneumoniae* infection. In addition, the increasing rate of macrolide-resistant strains and the harmful side effects of other sensitive antibiotics in young children make them difficult to treat and increase risks to health or of reinfection [16,17,18,19,20].

In recent years, multi-epitope vaccines have been discovered as a new strategy. Their antigen structure is stable and their conformation does not change. In addition, the use of multi-epitope vaccines can avoid toxic reactions caused by certain other subunit vaccines. Therefore, due to the advantages of high purity, strong specificity, and few side effects of epitope vaccines, the design and development of Mycoplasma pneumoniae epitope antigens are of great significance. The design of vaccines using epitopes is currently a focal point in the treatment of infectious diseases [21]. Epitope vaccines dynamically stimulate the immune system’s humoral and cellular branches. The vaccine is made up of highly immunogenic T cell epitopes, which activate the cytotoxic T lymphocyte (CTL) or helper T lymphocyte (HTL) to respond to specific epitopes [22]. Protective immune responses are stimulated significantly by HTL in many bacterial infections. Hence, recognizing the peptides responsible for T cell responses is vital for creating powerful epitope-based peptide vaccines [22]. Peptide vaccines that utilize epitopes may provide benefits such as affordable production, the ability to choose immune types, and better safety. Methods from immunoinformatics or computational biology have been crucial in designing peptide vaccines based on epitopes. Up to now, various types of *M. pneumoniae* vaccines have been reported, including whole cell vaccines, subunit vaccines and DNA vaccines [20]. However, the development of an *M. pneumoniae* vaccine has been difficult due to the continuous mutation of pathogens or autoimmune responses in early trials. Thus, it is necessary to design broad-spectrum vaccines for preventing large numbers of wild-type microorganisms. Thus, vaccine design based on conserved regions of antigens can effectively avoid the failure caused by pathogen mutations. In this study, we aim to design consensus sequences through the conserved regions of antigenic proteins from *M. pneumoniae* to extensively cover *M. pneumoniae* strains with various resources. The proposed in silico vaccine design strategy here for vaccines can be broadly applied as a promising candidate for pathogen-specific vaccines in treating pneumonia and the associated infections. The manuscript has been published in a preprint form [23].

## 2. Materials and Methods

### 2.1. Proteome Data and Consensus Sequences

Key structural proteins of *M. pneumoniae*, including HMW-1, HMW-2, HMW-3, and the p1 adhesin, were selected for predicting potential CTL and HTL epitopes. First, the proteins were de-duplicated through CD-HIT with 100% identification. Then, MAFFT v7.487 was used to perform multiple sequence alignment on these proteins. After further verification of these aligned sequences, the Python 3.7 script was used to identify their common regions, i.e., to retain the most frequently occurring amino acids, thus forming a consensus sequence. By aligning the genome sequences of different species or individuals, common conserved regions can be identified, which are often closely related to gene function.

### 2.2. Prediction and Evaluation of the MHC I and II Epitopes

The sites that bind to major histocompatibility complex class I (MHC I) with 9-mer and major histocompatibility complex class II (MHC II) with 15-mer were assessed through NetMHCIIpan 3.2 and NetMHCpan 4.1, respectively. We selected the corresponding Human Leukocyte Antigen (HLA) alleles based on the studies of Guan et al. (2025) [24]. For MHC I, the epitopes exhibiting the highest affinity to HLA (<0.5%) were chosen, while for MHC II, those epitopes displaying a comparatively lower affinity (<10%) were picked.

The VaxiJen v2.0, IEDB-tools, and ToxinPred were chosen for antigenicity prediction, epitope conservation analysis and toxic peptides prediction, respectively [24].

### 2.3. Immunogenicity of MHC I Peptides

The immunogenicity of MHC I peptides was predicted using the Class I Immunogenicity tool (http://tools.iedb.org/immunogenicity/, accessed on 19 February 2026) from the Immune Epitope Database (IEDB). It assesses the immunogenic epitopes by analyzing the physicochemical traits of amino acids and their sequence-specific localization. The peptides with positive predictions were likely to have immunogenicity, and we preserved the epitopes with scores exceeding 0.12.

### 2.4. Cytokine Induction of MHC II Peptides

Furthermore, the MHC II epitopes were evaluated by their capacity to induce the IFN-γ cytokine production. In this case, the IFNepitope server (https://webs.iiitd.edu.in/raghava/ifnepitope/design.php, accessed on 19 February 2026) was used to predict the characteristics of these epitopes with default parameters [24].

### 2.5. Population Coverage

To calculate population coverage, the T cell epitopes and their corresponding HLA binding alleles were used. The population coverage of each epitope was predicted using the online tool (http://tools.immuneepitope.org/population, accessed on 19 February 2026) from the IEDB server [25].

### 2.6. The Multi-Epitope Vaccine Construction

The multi-epitope vaccine (MEV) was constructed using epitopes that showed the best antigenicity and immunogenicity, as determined by the prior prediction and sequence filter. Specifically, the cholera toxin B subunit (GenBank: BBG62270.1) adjuvant was adjoined to the N-terminal with the “EAAAK” linker; the HTL epitopes were adjoined with the flexible linker “GPGPG”, while linear CTL epitopes were adjoined with the flexible AAY linker [24,26]. Using effective linkers can prevent protein misfolding and overlap of functional regions. For example, the GPGPG linker refers to a peptide linker composed of repeating glycine-proline sequences. In vaccine design, the choice of this linker is based on two key aspects: Firstly, glycine (abbreviated as G) offers high flexibility, allowing the linked protein structures to fold freely and reducing steric hindrance. Secondly, proline (abbreviated as P) imparts rigidity to the linker, maintaining structural stability. The alternating GPGPG sequence ensures that the spatial conformation of vaccine epitopes remains unaffected while enhancing their stability, thereby promoting proper folding and presentation of epitopes and improving vaccine immunogenicity. Therefore, we employed the GPGPG linker to connect different antigenic epitopes in the vaccine design to optimize its structure and function.

### 2.7. Assessment of Physicochemical Properties and Solubility

The physicochemical traits of proteins are significant characteristics. The physicochemical characteristics of the predicted epitopes were analyzed using Expasy Protparam (https://web.expasy.org/protparam/, accessed on 19 February 2026) to gain further insight into the basic nature of the vaccine. In addition, the solubility of the vaccine was analyzed through the Protein-Sol website (http://protein-sol.manchester.ac.uk, accessed on 10 February 2026).

### 2.8. Antigenicity and Allergenicity Analysis

Further research was conducted on the peptides to verify their potential for inducing allergies and immunogenicity. For antigenicity prediction, we used VaxiJen v2.0 (threshold ≥ 0.4) to determine the ability of the multi-epitope to elicit an immune response, and the AllerTop v2.0 (https://www.ddg-pharmfac.net/AllerTOP/, accessed on 10 February 2026) was used to evaluate its allergic properties.

### 2.9. Secondary Structure and Three-Dimensional Structure Optimization and Verification

The secondary structure of the designed vaccine was predicted on the PSIPRED server (http://bioinf.cs.ucl.ac.uk/psipred/, accessed on 10 February 2026), which provided a range of protein structure prediction methods. The AlphaFold 3.0 server (https://aideepmed.com/I-TASSER/, accessed on 10 February 2026) was used to predict the three-dimensional (3D) structure of the vaccine that was developed. Afterwards, the identified 3D structure underwent refinement via the GalaxyRefine web server (http://galaxy.seoklab.org/refine, accessed on 10 February 2026). We identified the selected structure based on the lowest energy score and the highest RMSD value. The structure was visualized using PyMOL v2.5.7 following optimization and identification. Furthermore, the refined 3D model of the designed vaccine was accessed through the Rampage website (https://saves.mbi.ucla.edu/, accessed on 10 February 2026) and the ProSA website (https://prosa.services.came.sbg.ac.at/prosa.php, accessed on 10 February 2026), aiming to analyze the deviations from the average [24,26].

### 2.10. Disulfide Engineering of the Designed Vaccine

Disulfide bonds usually play a crucial part in stabilizing the protein’s folded state by reducing its structural disorderliness and enhancing its energetic stability. In order to enhance stability, the Disulfide by Design 2.0 website (http://cptweb.cpt.wayne.edu/DbD2/) was used to predict the disulfide bonds of the constructed vaccine (accessed on 21 February 2025). The structure was used to scrutinize potential cysteine mutations, which resulted in the creation of disulfide bonds within the vaccine. The energy of residue pairs ≤ 2.2 kcal/mol was selected as the threshold [24].

### 2.11. *Molecular Docking of Model Proteins to TLRs*

Molecular docking was used to explain how model proteins bind with receptor molecules. As viral glycoproteins and dsRNA can always identify the toll-like receptors TLR4 (PDB ID: 2Z63) and TLR3 (PDB ID: 1ZIW), the two receptors were chosen as the immunological receptors. In this case, the refined model was submitted as a ligand for molecular docking with ClusPro v2.0 (https://cluspro.bu.edu/, accessed on 19 February 2026). Before docking, water molecules in the ligand and receptor were removed, while hydrogen ions were added.

### 2.12. Normal Mode Analysis of the Designed Vaccine

The iMODS tool (http://imods.chaconlab.org/, accessed on 19 February 2026) was used for normal mode analysis to examine the stability and physical movements of the vaccine–receptor docked complexes. This tool can use normal mode analysis (NMA) in the inner coordinates to predict the collective motions of proteins. The IMODS tool calculates the deformability, eigenvalues, variance, covariance plot, B-factor, and elastic network of vaccine–receptor complexes.

### 2.13. In Silico Vaccine Cloning

Achieving the maximum effect of codons, as optimized previously, can boost the production of vaccine antigens in the host. The EMBOSS Backtranseq (https://www.ebi.ac.uk/jdispatcher/st/emboss_backtranseq) was utilized to translate the vaccine protein in reverse (accessed on 24 February 2025). The JCat website (http://www.jcat.de/) was utilized to obtain the level of protein expression (accessed on 24 February 2025). We chose the *Escherichia coli* K12 as the organism of host expression. We inferred the protein expression level through the CAI (codon adaptation index) and the GC content percentage. As reported, a score ≥ 0.8 generally signified a satisfactory score for CAI, and the GC% was between 30 and 70%, which meant that the efficiency of gene translation and transcription was high [27].

### 2.14. Experimental Animals and Cell Lines

The 6–8 week old female BALB/c mice were purchased from Beijing Vital River Laboratory Animal Technology Co., Ltd. (Beijing, China). All animal studies that do not involve pathogenic pathogens are conducted in SPF-level facilities. A total of six mice were randomly divided into the treatment group (T cell epitope peptide immunization group) and the control group (PBS group), with three mice in each group. HEK-293 cells were cultured in growth medium (Dulbecco modified Eagle medium containing 10% fetal bovine serum and antibiotics) and incubated at 37 °C and 5% CO_2_.

### 2.15. Mice Immunization and Detection of Specific Antibody Levels

We constructed and synthesized recombinant plasmid pET-29a-MP based on the designed sequence. Then, we transformed the recombinant plasmid into competent cells of *E. coli* BL21 (DE3) and constructed the recombinant strain pET-29a-MP. The pET-29a-MP and Freund’s adjuvant were mixed in a 1:1 ratio and thoroughly vortexed to prepare the pET-29a-MP subunit vaccine.

The mice were divided into two groups and labeled respectively. The mice in the treatment group were given 30 μg vaccine and Freund’s adjuvant by subcutaneous injection into the back, while the mice in the control group were given a placebo (PBS). Notably, the prime immunization was performed with complete Freund’s adjuvant (CFA), followed by booster immunizations with incomplete Freund’s adjuvant (IFA). Immunization was administered twice before and after, with a one-week interval between each immunization (day 0 and day 7), and samples were collected uniformly on day 14. At the end of the experiment, mice were euthanized using the cervical dislocation method, and blood was collected from the eyeballs.

### 2.16. Serum Cytokine and Antibody Analysis

Blood samples were collected via orbital venous plexus bleeding from three mice per group at 14 days post-vaccination. After standing overnight at 4 °C, they were centrifuged for 6 min at 4000 rpm for serum separation and stored at −80 °C. The serum was used for measurements of IgG, IgG1, IgG2a, IFN-γ, IL-2, IL-4 and IL-10 using commercial chicken ELISA kits (Jiangsu Jingmei Biotechnology Co., Ltd., Yangcheng, China) using the protocol provided by the manufacturer.

## 3. Results

### 3.1. The Process of Vaccine Design

**Figure 1:**
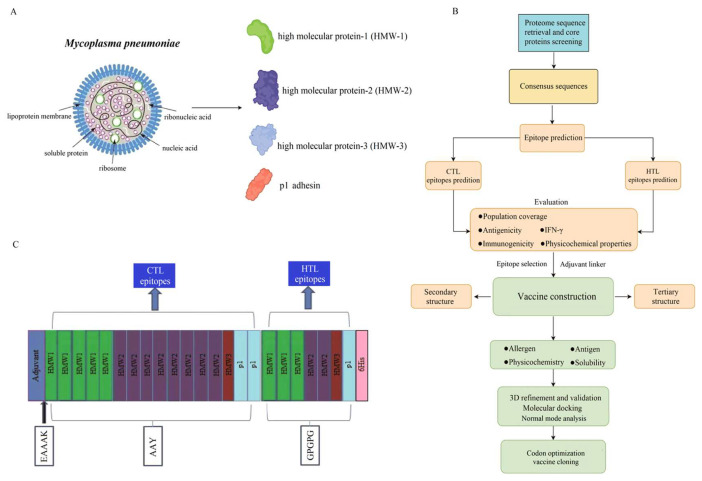
The pipeline of the Mp vaccine construction. (**A**): The structural proteins of *M. pneumoniae*. (**B**): The process of constructing the MEV. (**C**): The candidate T cell vaccine was structurally designed by combining CTL and HTL epitopes with the C-terminus of the vaccine being added with 6 × His (histidine).

Antigenic peptide vaccines consist of multiple T cell epitopes. The structural proteins, including HMW1 (*n* = 10), HMW2 (*n* = 10), HMW3 (*n* = 10) and p1 adhesin protein (*n* = 4), were selected and downloaded from the National Center for Biotechnology Information (NCBI) (Figure 1A), and the vaccine design process is shown in Figure 1B. Initially, the HMW1–3 and p1 adhesin proteins were chosen and aligned to maintain sequence consistency. The generated consensus sequence showed 99.71%, 99.94%, 99.85% and 96.46% sequence similarity with the reference proteins HMW1 (WP_010874803.1), HMW2 (WP_010874666.1), HMW3 (WP_010874808.1) and p1 adhesin (WP_010874498.1), respectively. Then, the consensus sequences were chosen to predict the putative T cell epitopes for the vaccine design. The vaccine was constructed by sequentially linking CTL epitopes followed by HTL epitopes, using appropriate peptide linkers to ensure structural integrity and immunogenic presentation. The adjuvant had to be connected at the N-terminus, and AAY linkers were used to connect the CTL epitopes, while GPGPG linkers were used to connect the HTL epitopes (Figure 1C), which were then used for further vaccine evaluation.

The IEDB website (http://tools.iedb.org/, accessed on 20 September 2025) was utilized to identify potential CTL and HTL epitopes from the structural proteins of *M. pneumoniae*. A total of 350 CTL epitopes with 9-mer core sequences were identified in the chosen HMW1–3 and p1 adhesin proteins. The choice of immunogen or epitope is the initial step for effective vaccine design; henceforth, to find out the most probable antigenic protein, the protein sequences of *M. pneumoniae* were retrieved for dataset construction. Further evaluation revealed that 16 optimal CTL epitopes showed high antigenicity, immunogenicity, non-toxicity and non-allergenicity, and these were selected for the final vaccine development (Table 1). Similarly, a total of 1271 HTL epitopes with 15-mer core sequences were identified by using the IEDB server. Among them, only seven optimal HTL epitopes triggered the production of IFN-γ, which was selected for the ultimate vaccine design, as shown in Table 1. Finally, with the N-terminal adjuvant, the constructed vaccine comprised 458 amino acids, which was designed based on 23 epitopes, including 16 CTL epitopes and 7 HTL epitopes, respectively (Table 1; Figure 1B).

### 3.2. % Percentile Rank: MHC I < 0.5%, MHC II < 10%3.2 Global Population Coverage

**Figure 2:**
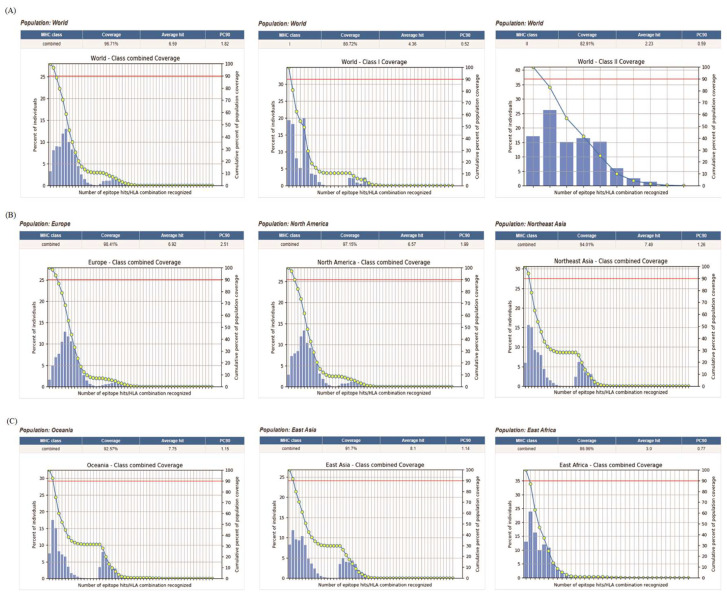
The population coverage of the CTL and HTL epitopes. (**A**) The global distribution and coverage of total epitopes, HTL epitopes and CTL epitopes. (**B**) The regional population coverage of Europe, North America and Northeast Asia. (**C**) The regional population coverage of Oceania, East Asia and East Africa. The 90% red line represents that the cumulative population coverage reaches 90%. It is used to measure the extent to which the immune epitopes induced by the vaccine can cover the target population, and to assist in evaluating the protection scope and potential of the vaccine for the population. It serves as a reference standard for the immune epitopes related to the vaccine to enable 90% of the target population to generate immune recognition and response.

The chosen CTL and HTL epitopes were assessed with regard to population coverage, as shown in Table 1. As a whole, 96.71% population coverage of the CTL and HTL epitopes was observed worldwide. The global average population coverage of CTL epitopes was 80.72%, while that of HTL epitopes was 82.91% (Figure 2A). The selected epitopes engaged with numerous HLA alleles across various geographical areas, including North America (97.15%), Oceania (92.57%), East Asia (91.7%), Northeast Asia (94.01%), Europe (98.41%) and East Africa (86.96%) (Figure 2B,C). The high global population coverage indicates that the designed vaccines with these epitopes may be effective for the majority of the global population.

### 3.3. Physicochemical Properties and Immunological Evaluation

Furthermore, we assessed the vaccine’s physical and chemical characteristics. The results showed that the molecular weight of the vaccine was 50,110.47 Da, the antigenicity score was 0.62 (reference value: 0.4), and the immunogenicity score was 5.96, indicating a pronounced antigen and considerable immunogenicity (Table 2). Moreover, other characteristics were assessed, such as the pI of the theoretical isoelectric point (5.34), the index of instability (31.46), the aliphatic index (76.29), the grand average of hydropathicity (−0.22), and the scaled solubility (0.34), revealing high solubility and hydrophilicity (Table 2).

### 3.4. Secondary and 3D Structure: Verification and Optimization

**Figure 3:**
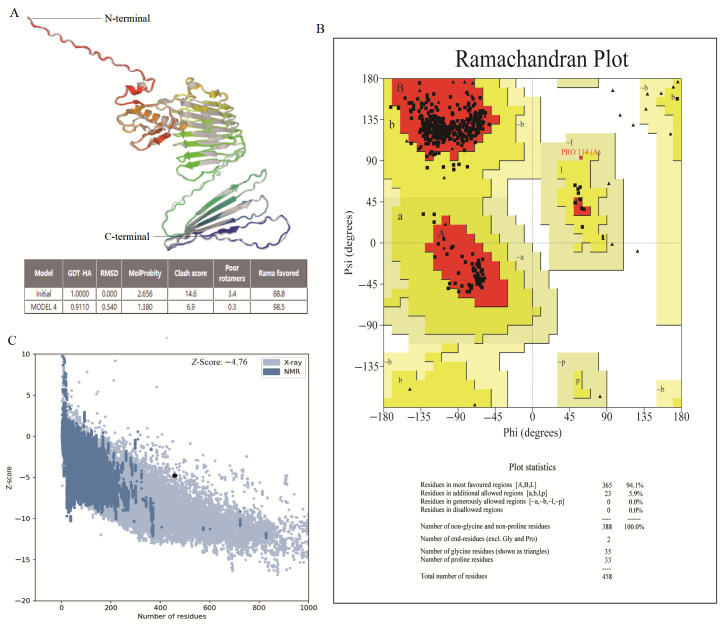
The validation of the tertiary structure of the MEV. (**A**) The optimized 3D structure of the MEV. (**B**) The statistics of the Ramachandran plot, indicating the most acceptable, disallowed and favorable regions. (**C**) The ProSA-web result, with a *Z*-score of −4.76 for the optimized vaccine model.

The secondary structure of the vaccine—the β-strand, α-helix and random coils—was showed on the PSIPRED server. The results showed that its secondary structure comprised 46.07% (211/458) α-helix, 12.23% (56/458) β-strand, and 41.7% (191/458) coils (Table 2). Furthermore, the 3D structure of the vaccine was refined for assessment and verification (Figure 3A). The Ramachandran plot for the improved vaccine model showed that 94.1% of the residues were in favorable regions, 5.9% were in allowed regions, and none were in non-allowed regions (Figure 3B). Similarly, the overall quality of the model was indicated by the indicator of the *Z*-score generated by the ProSA server. The area of NMR (Nuclear Magnetic Resonance) spectroscopy and X-radiation was similar to the *Z*-score, pointing to outstanding structural quality. The results showed that the *Z*-score of the refined model was −4.76 (Figure 3C), corresponding more closely to the areas generated by NMR and X-rays.

### 3.5. Disulfide Engineering

**Figure 4:**
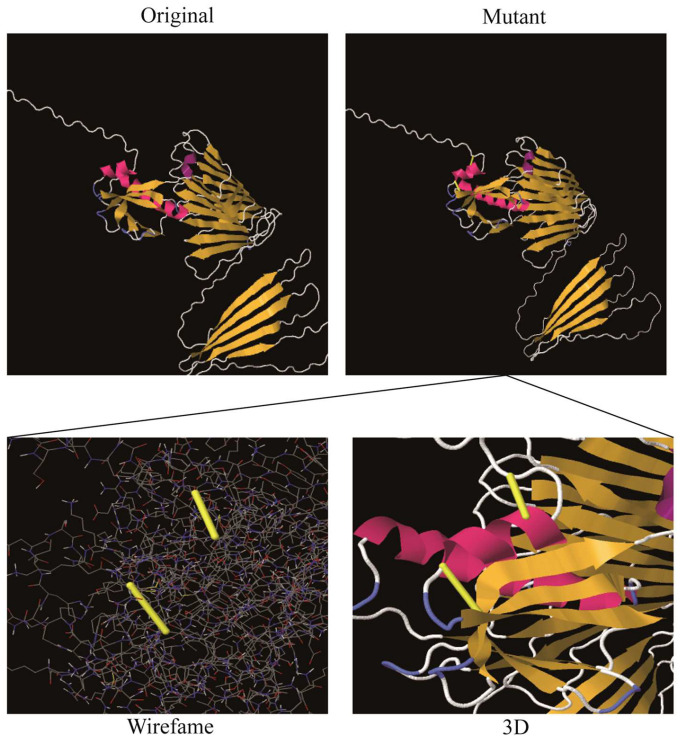
Disulfide bond design in the MEV. The mutant variant features Pro23 and Asp28 and Tyr33 and Thr36 (two pairs of amino acids) that have been mutated into cysteine residues, which form disulfide bonds represented by yellow sticks.

Disulfide bonds enhance the stability of numerous extracellular and secreted proteins by reducing their conformational entropy and increasing the free energy of their denatured state, thereby stabilizing their folded forms [28]. Leaning on disulfide bonds established between the residues, the stability of the vaccine’s structure was maintained. After we investigated using the Disulfide by Design 2.0 website (http://cptweb.cpt.wayne.edu/DbD2/, accessed on 20 December 2025), the result indicated that the vaccine construct contained 9 potential residue pairs that had the potential to form disulfide bonds, as shown in Table 3. The pairs of Pro23 and Asp28 (residue pair and 1.96 kcal/mol) and Tyr33 and Thr36 (residue pair and 1.34 kcal/mol) could be mutated with the lowest threshold scores (<2.2 kcal/mol) (Figure 4) [28].

### 3.6. Molecular Docking

**Figure 5:**
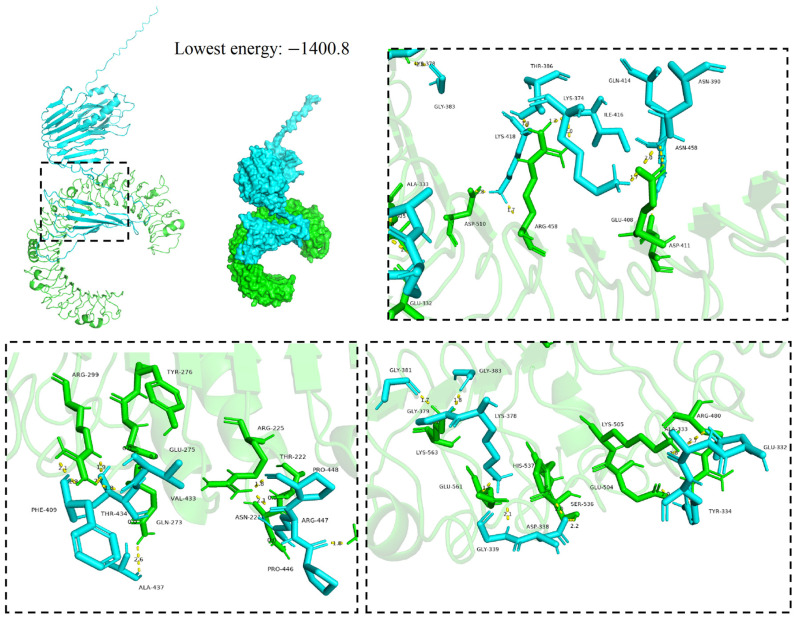
Molecular docking of MEV with TLR3 molecules; the model with the lowest energy was selected as the optimal binding mode. Additionally, molecular docking of the MEV with TLR3 molecules is shown in a three-dimensional diagram. The yellow dashed lines indicate hydrogen bonds.

**Figure 6:**
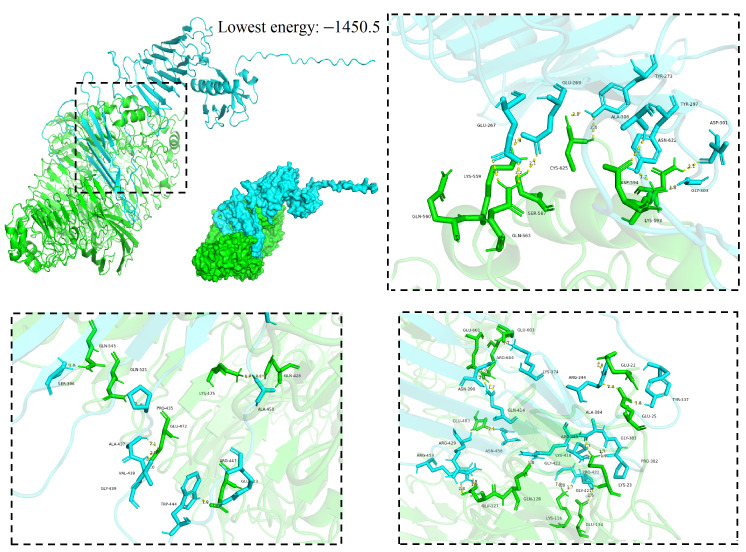
Molecular docking of MEV with TLR4 molecules; the model with the lowest energy was selected as the optimal binding mode. Additionally, molecular docking of the MEV with TLR4 molecules is shown in a three-dimensional diagram. The yellow dashed lines indicate hydrogen bonds.

The vaccines served as ligands for molecular docking with the TLR3/4 receptors to predict their interactions and binding affinities. In this scenario, 30 docked complexes with various poses were generated by the ClusPro (version 2.0) server. We performed molecular docking of the designed vaccine with TLR3 and of the TLR4 agonist (50S ribosomal protein L7/L12) with TLR4. Cluster 12 from the vaccine–TLR3 docking clusters and cluster 8 from the vaccine–TLR4 docking clusters were selected with excellent comprehensive evaluations (hydrogen bonding, van der Waals forces, electrostatic interactions), respectively. Furthermore, an analysis was conducted on the binding interactions of the selected complex, as well as an exploration of its involvement in active site residues. The interplay between 30 hydrogen bonds was displayed by the vaccine–TLR3 compound in the interaction plane. The Thr386, Lys374, Lys418, Glu332, Asn458, Phe409, Thr434, Ala437, Arg447, Pro448, Gly381, Gly383, His537, Gly339, Asp338, and Tyr334 residues of the vaccine were able to form hydrogen bonds with the Arg458, Asp510, Lys378, Glu408, Asp411, Arg299, Gln273, Arg225, Gly379, Glu561, Ala333, Ser536, and Glu504 residues of the TLR3 receptor (Figure 5). Similarly, the interplay between 35 hydrogen bonds was displayed by the vaccine–TLR4 compound in the interaction plane. The Glu269, Glu267, Tyr273, Asn622, Ala308, Asp301, Gly303, Lys374, Gln414, Asn458, Arg429, Arg453, Gly423, Gly421, Lys418, Arg385, Gly383, Arg344, Tyr337, Ala450, Ser396, Ala437, Val438, and Trp444 residues of the vaccine were able to form hydrogen bonds with the Ser587, Lys559, Gln563, Cys625, Lys593, Asp594, Glu603, Gly601, Glu483, Glu127, Glu128, Glu134, Lys116, Pro422, Glu22, Glu25, Gln428, Lys475, Gln545, Glu472, and Arg447 residues of the TLR4 receptor (Figure 6).

In addition, benchmarking showed that in the vaccine–TLR3/4 complexes, the lowest binding energy between the vaccine and TLR3 receptor and the vaccine and the TLR4 receptor was −1400.8 and −1450.5, respectively (Figure 5 and Figure 6). In contrast, the lowest binding energy between the glycoprotein and TLR3, and between it and TLR4 was −1105 and −1027, respectively, indicating that the vaccine–TLR3/4 compounds had a more compact conformation and stable binding interaction.

### 3.7. Normal Mode Analysis

**Figure 7:**
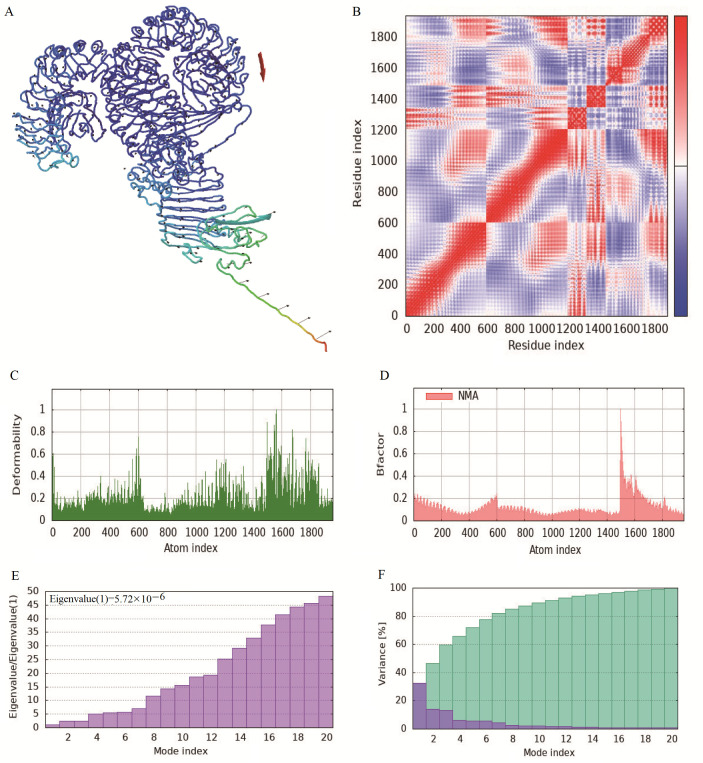
Normal mode analysis of the vaccine–TLR4 complex. (**A**) Affine-arrows of the vaccine-TLR4 complex. (**B**) Covariance map of the complex. Covariance matrix indicates coupling between pairs of residues, i.e., whether they experience correlated (red), uncorrelated (white) or anti-correlated (blue) motions. (**C**) Deformability index; (**D**) B-factor column; (**E**,**F**) Eigenvalue and NMA variance.

The normal mode analysis (NMA) of iMODS was used to check the stability and motion of docking complexes. In this study, using NMA, the slow kinetics of docking complexes and their large amplitude conformational changes were further analyzed. The results showed that the receptor and ligand tended to cluster together (Figure 7A), and the covariance map showed that the binding region covered many red colors, indicating that the key region of the protein had coordinated amino acid movement and stable ligand binding (Figure 7B). The deformability built up the independent distortion of each residue, portrayed by the method of chain hinges (Figure 7C). The overall binding peak of the vaccine–TLR4 complex is moderate, indicating a certain degree of flexibility, but it is not excessively twisted, which is a good manifestation of docking results. After docking, most of the B-factor fluctuated within a reasonable range (<0.2) without affecting the natural movement pattern of the protein (Figure 7D). The eigenvalue, which is a crucial parameter of a stable structure, must be high to have a stable complex. The eigenvalue determined for the complex was found to be 5.72 × 10^−6^, with the variance of each typical mode being gradually decreased (Figure 7E,F). These rates are significantly higher for structural stability. The moderate eigenvalues indicate that the protein maintained its biological activity after binding and adapted to ligand binding.

### 3.8. Codon Optimization and Vaccine Cloning

**Figure 8:**
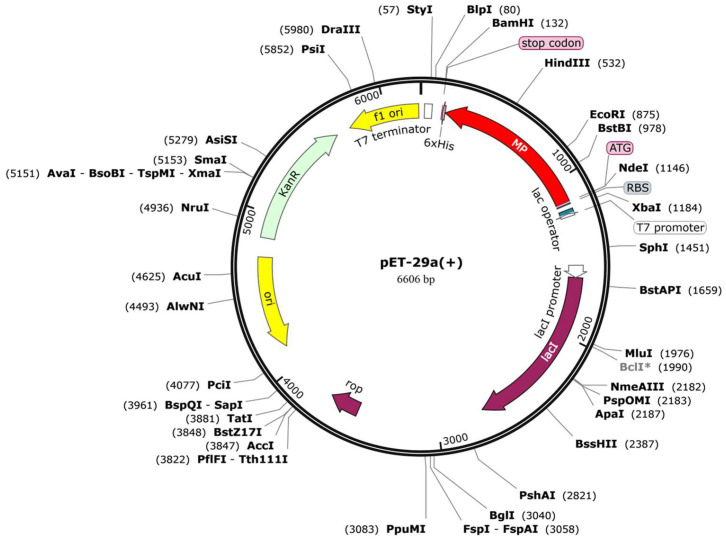
In silico cloning of the vaccine construct sequence into the pET-29a(+) expression vector. The recombinant product, with the vaccine construct inserted and highlighted in red within the vector, is shown. The expression system of *E. coli* was used to express the *M. pneumoniae* vaccine.

To ensure high-level expression and ease of production of vaccines, codon optimization is performed through JCat servers. The cDNA length of the designed vaccine is 1374 bp, with a stop codon added at the end of the cDNA. The CAI value (score: 1) and GC content (53.42%) of the vaccine are both ideal, indicating the possibility of high gene expression potential and excellent expression ability in the type strain *E*. *coli* K12. Finally, the cDNA sequence of the vaccine is cloned into the pET-29a(+) vector with SnapGene v5.2.3 (Figure 8). In the pET-29a(+) vector, the T7 promoter and T7 terminator are like a “switch” and a “brake”. The gene inserted between them is the vaccine-related sequence (marked as MP in the figure) that we want to express. It will be transcribed under the drive of the T7 promoter and ultimately used for subsequent vaccine synthesis.

### 3.9. Initial Immune Induced Humoral and Cellular Immune Responses

**Figure 9:**
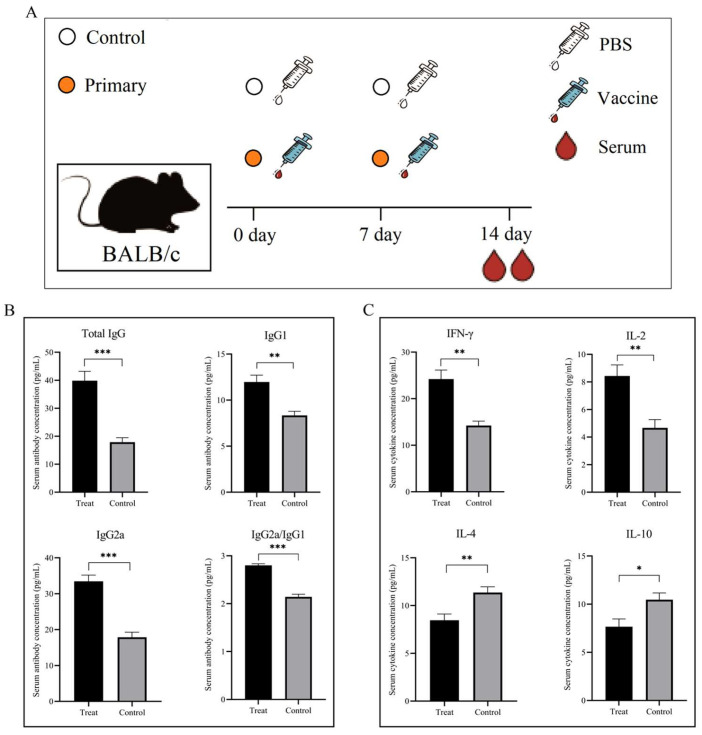
Evaluation of systemic immune responses following prime-boost vaccination in BALB/c mice. (**A**) Experimental schema of the immunization protocol. Mice received injections on day 0 (prime vaccination) and day 7 (boost vaccination). Serum samples were collected from all mice on day 14 for subsequent immunological analyses. (**B**) Vaccine-induced serum antibody responses. The treated group (Treat, black bars) shows significantly elevated levels of Total IgG, IgG1, and IgG2a compared to the control group (gray bars). Asterisks indicate statistically significant differences (** *p* < 0.01, *** *p* < 0.001). (**C**) Serum cytokine profiles indicating Th1 polarization. The treated group (Treat, black bars) exhibited significantly higher production of the Th1-associated cytokines IFN-γ and IL-2 compared to controls. Conversely, the concentrations of Th2-associated cytokines, IL-4 and IL-10, were significantly lower in the treated group than those in the control group. Asterisks indicated statistically significant differences (* *p* < 0.05, ** *p* < 0.01).

In this study, we evaluated the humoral and cellular immunogenicity of MEV, using ELISA to detect the IgG antibody and different cytokine levels in the serum (Figure 9A). As shown in Figure 9B, after vaccination, the analysis of serum antibody levels showed that compared with the control group, the mice in the treatment group had significantly increased levels of Total IgG, IgG1, and IgG2a in their serum. The serum IgG2a/IgG1 ratio was significantly higher in the treatment group compared to the control group (*p* < 0.001), indicating that this vaccine more effectively promotes IgG2a production and enhances a Th1-type immune response. In addition, compared with the control group, the levels of serum IFN-γ and IL-2 in the treatment group were significantly increased, while the levels of IL-4 and IL-10 were significantly decreased (Figure 9C). The preliminary experimental results above indicate that the *M. pneumoniae* vaccine designed in this study had a good immune effect and the overall vaccine design was reasonable.

## 4. Discussion

*M. pneumoniae* is a major cause of lower respiratory tract infections in both newborns and older people with weakened immune systems. *M. pneumoniae* and the widely reported foodborne pathogens are both high-threat pathogens that WHO is focused on preventing and controlling in the global disease burden, and have a significant impact on public health [1,29,30]. Currently, there is no effective vaccine for treating Mp infection. Contemporary immunoinformatics tools offer a practical and efficient alternative to traditional vaccine development, providing a less labor-intensive screening method [31,32]. Vaccines have consistently demonstrated high safety and efficacy in preventing infectious diseases, offering acquired immunity against diseases [33,34]. An ideal target should be very conservative, capable of inducing neutralizing cellular immunity and producing antibodies against *M. pneumoniae*, which is important for the development of effective pneumonia vaccines. The membrane-associated proteins and cell adhesion proteins of *M. pneumoniae* are extensively produced during disease progression and exhibit strong conservation and immunogenicity, making them the optimal immunogens for triggering humoral and cell-mediated immune responses. Due to the continuous mutations of wild-type viruses, this study aimed to develop a T cell epitope-based vaccine, providing cross-protection against various wild-types. If T cell epitopes have are well-conserved in the selected protein, they are considered strong and effective. Although vaccines have the potential to act as a preventive tool for future epidemic occurrences, developing effective broad-spectrum vaccines currently remains a challenging task. Thus, it is clear that a novel approach to vaccine development is necessary to find solutions to pressing public health threats. *M. pneumoniae* encodes multiple virulence factors, including adhesins, glycolipids, toxic metabolites, community-acquired respiratory distress syndrome (CARDS) toxins, and capsular polysaccharides. Among these virulence factors, the HMW1–3 and p1 adhesin and other adhesion-related proteins are important antigens in *M. pneumoniae* that have immunogenicity and immunoreactivity and can induce specific neutralizing antibodies [13]. Given the established roles of HMW1–3 and p1 adhesin proteins as critical determinants of immune evasion and interhuman transmission, this investigation prioritized the engineering of a multi-epitope vaccine construction [13,35,36]. This involved generating consensus sequences of the target structural proteins through rigorous multiple sequence alignment (MSA) to maximize coverage of evolutionarily conserved residues across prevalent clinical isolates, thereby targeting immunodominant epitopes with high population coverage frequencies. Furthermore, we aimed to identify the surface antigen regions of antigens that facilitated recognition by the humoral and cellular immune systems. Here, we employed an immunoinformatic-driven approach to screen for significant dominant immunogens against *M. pneumoniae*, and the results indicated that all membrane-bound proteins and cell adhesion proteins had good antigenicity, with the highest antigen scores. All possible epitopes of HTL and CTL should be identified and assessed at the initial step. The elimination of the amount of virus present depended on a response of the cell (CD8+ T) initiated by the host. The research relating to the cells (CD8+ T) infected between humans and mice with *M. pneumoniae* was scarce [37,38]. The epitopes that triggered strong and effective CTL responses deserve thorough study and are promising candidates for antiviral vaccine development. The evaluation including epitopes (HTL and CTL) and required linkers was carried out using an integrated ranking process of the final vaccine. The process of developing the vaccine was crucial in order to enhance the expression of profiles, the folding properties of the vaccine, and stability. The vaccine’s stability and durability were increased by linking the adjuvants and epitopes of CTL via the EAAAK linkers, which assisted in eliciting strong cellular and humoral immunity targeting particular antigens [39,40]. Beyond its established importance as a key physicochemical property of recombinant vaccines [41], solubility is also a parameter that can be assessed computationally. Here, the physicochemical properties prediction showed that the vaccine exhibited an acidic theoretical pl of 5.34, which was distinct from physiological pH and may favor solubility under such conditions. On the other hand, the vaccines could withstand high temperatures and were hydrophilic, as shown by the GRAVY value and the lipids index. The predicted favorable physicochemical properties and positive parameter scores of the designed vaccine indicated a high likelihood of being an effective candidate against infections.

Using the methods in this study, the global coverage rate of the best population reached 96.71%; thus, the designed vaccine will likely become a highly promising competitor. The Ramachandran plot was used to evaluate the rationality of protein conformation. It does not consider energy issues and only evaluates whether the conformation is reasonable. The evaluation of the model quality after homology modeling shows that the proportion of amino acid residues falling within the allowed and maximum allowed regions is over 94%, indicating that the conformation of the model was reasonable. In addition, after molecular docking, the peptide vaccine showed potential for inhibiting infection via its interaction with TLR3 and TLR4, both favorable immune receptors. The vaccine model may act as a ligand by means of a significant interaction occurring on the surface of the TLR3/TLR4 receptors. Furthermore, the vaccine–TLR4 docked complex was also subjected to normal mode analysis to determine its stability. The results revealed that the vaccine can adapt to the ligand without drastic fluctuations, showing a stable binding force. The CAI value and GC content were used to evaluate the effectiveness of the vaccine development process. The designed vaccine is predicted to be well-expressed in the system of *E. coli* K12.

The MEV designed for *M. pneumoniae* in this study has many advantages compared to the available designed *M. pneumoniae* vaccines. First, in terms of antigen selection, Mahmood et al. (2021) [42] and Shahbazi et al. (2025) [26] selected highly antigenic proteins from single strains, such as *M. pneumoniae* ATCC 29342/M129 and *M. pneumoniae* M129-B7; however, in this study, we chose four important structural proteins (HMW1–3, p1-adhesive protein) from different strains of *M. pneumoniae* as antigens and generated conserved consensus sequences respectively to ensure a broad antigen coverage. It is worth noting that the vaccine designed in this study has a high solubility (0.341), which is close to the solubility of the vaccine designed by Vilela Rodrigues et al. (2022) (0.383) [38]. Meanwhile, the molecular weight of the vaccine designed in this study is lower than that of those designed by Mahmood et al. (2021) and Vilela Rodrigues et al. (2022) [38,42]. Second, the immunogenicity scores of seven selected CTL epitopes by Mahmood et al. (2021) [42] were very low (0.02–0.38, average score: 0.121), while the CTL epitopes in this study were predicted to have high immunogenicity (0.2–0.4, average score: 0.238). Meanwhile, the vaccine in this study has high antigenicity (0.62) and high immunogenicity (5.96), higher than those of the vaccines designed by Vilela Rodrigues et al. (2022) (antigenicity: 0.53) [38], but slightly lower than that of the vaccine designed by Shahbazi et al. (2025) (antigenicity: 0.86) [26]. Third, most of the HTL epitopes selected by Mahmood et al. (2021) [42] were predicted to have no capacity of inducing the synthesis of IFN-γ, while the HTL epitopes in this study were predicted to be able to induce IFN-γ production. Fourth, in terms of population coverage assessment, the MEV designed by Mahmood et al. (2021) [42] only had a 83.12% worldwide population coverage, and 50.69% worldwide coverage was achieved by Shahbazi et al. (2025) [26], while the vaccine designed in this study had a 96.71% worldwide population coverage. Fifth, in the 3D structure evaluation of MEV, only 71% of the residues in the MEV designed by Mahmood et al. (2021) [42] were located in favored regions, and 87.3% in favored regions by Vilela Rodrigues et al. (2022) [38]; however, in this study, 94.1% of the residues were located in the favored regions. Last but not least, during the docking process with TLR4 receptors, the vaccine designed in this study had a lower binding energy than that of Mahmood et al. (2021) [42]. In addition, identifying the specific sites of disulfide engineering in the vaccine designed in this study provides a clear and feasible strategy for further improving its stability, a feature that has not been detailed in other studies [26,38,42]. In summary, the MEV designed here is significantly superior to that designed by Mahmood et al. (2021) [42] in terms of immunogenicity, population coverage, physicochemical properties, and protein conformation, and is a potential candidate vaccine for *M. pneumoniae* infection. Preliminary mouse immunization experiments have confirmed that the designed *M. pneumoniae* multi-epitope vaccine could effectively induce Th1-type immune responses, inhibit Th2-type responses, and possibly provide protective immune characteristics against *Mycoplasma* infection. In contrast, Mahmood et al. (2021) [42], Vilela Rodrigues et al. (2022) [38] and Shahbazi et al. (2025) [26] lack preliminary validation, which compromises the persuasiveness of their proposed vaccines..

## 5. Conclusions

Immunogenic epitope analysis provided a crucial insight that guided the creation of vaccines that were both safe and effective, capable of triggering strong and wide-ranging immune responses. In this study, the consensus sequences were used to predict potential T cell epitopes from structural proteins, ensuring the breadth and conservation of the antigens. The broad-spectrum vaccine designed here has immunological advantages, wide population coverage, and the ability to effectively interact with TLR3 or TLR4 immune receptors, which may result in a strong immune response after viral infection. The designed multi-epitope vaccine demonstrates structural stability, high solubility, strong immunogenicity, and non-toxicity, and has been preliminarily validated to be reasonable through experiments, representing a promising candidate for further development against *M. pneumoniae*.

## Figures and Tables

**Table 1:** List of the HTL and CTL epitopes selected to construct the candidate vaccine.

Types	Protein	GRAVY	Peptides	Length (mer)	Location	Alleles	Antigenicity Score	Class I Immunogenicity	IFN-γ Score	Population Coverage
CTL	HMW1	−0.04	SLDPIGETA	9	256–265	HLA-A*02:01, HLA-C*01:03, HLA-C*01:02	0.87	0.26	ND	45.57%
		−0.24	LQPEPVTEV	9	290–299	HLA-A*02:01, HLA-C*01:03, HLA-C*01:02	1.14	0.22	ND	45.57%
		0.56	TIAEITPQV	9	326–335	HLA-A*26:01, HLA-C*01:03, HLA-C*01:02	0.98	0.2	ND	15.84%
		0.22	AINFDDIFK	9	746–755	HLA-A*11:01, HLA-C*01:03, HLA-C*01:02	0.47	0.34	ND	24.52%
		−1.02	KLDDFDFET	9	982–991	HLA-A*02:01, HLA-C*01:03, HLA-C*01:02	1.81	0.32	ND	45.57%
	HMW2	−0.9	SRYANWADF	9	133–142	HLA-B*27:05, HLA-C*01:03, HLA-C*01:02	1.89	0.29	ND	14.92%
		−1.26	KRREIDDLL	9	421–430	HLA-B*27:05, HLA-C*01:03, HLA-C*01:02	1.13	0.28	ND	14.92%
		−0.1	FLEGEFNHL	9	590–599	HLA-A*02:01, HLA-C*01:03, HLA-C*01:02	0.61	0.29	ND	45.57%
		−0.37	ASKERILDF	9	738–747	HLA-B*08:01, HLA-C*01:03, HLA-C*01:02	1.08	0.21	ND	20.08%
		0.31	TEELEAAFL	9	836–845	HLA-B*40:01, HLA-C*01:03, HLA-C*01:02	0.76	0.26	ND	17.63%
		0.84	ELKIAFADL	9	919–928	HLA-B*08:01, HLA-C*01:03, HLA-C*01:02	1.92	0.26	ND	20.08%
		−1.43	NLAEREREI	9	1539–1548	HLA-B*08:01, HLA-C*01:03, HLA-C*01:02	1.43	0.36	ND	20.08%
		−0.9	YPYPYPWFY	9	1622–1631	HLA-A*01:01, HLA-C*01:03, HLA-C*01:02	0.95	0.23	ND	26.14%
	HMW3	0.98	APVVEPTAV	9	287–296	HLA-B*07:02, HLA-C*01:03, HLA-C*01:02	0.63	0.2	ND	22.06%
	p1	−0.2	KADDFGTAL	9	334–343	HLA-B*39:01, HLA-C*01:03, HLA-C*01:02	0.9	0.23	ND	13.10%
		−0.08	YVPWIGNGY	9	811–820	HLA-A*26:01, HLA-C*01:03, HLA-C*01:02	0.53	0.4	ND	15.84%
HTL	HMW1	−1	DYLQYVGNEAYGYYD	15	105–120	HLA-DRB1*04:01, HLA-DRB1*09:01	0.49	ND	0.91	17.24%
		−0.97	RSLSNDFTIAHRPSD	15	825–840	HLA-DRB1*03:01	0.81	ND	0.47	17.84%
		−0.37	KNIQITLKELKAVYK	15	866–881	HLA-DRB1*03:01, HLA-DQA1*01:01/DQB1*02:01, HLA-DQA1*01:01/DQB1*03:01	1.37	ND	0.47	67.86%
	HMW2	−0.54	ARTQFDNRVSLLSAR	15	608–623	HLA-DRB1*03:01	1.21	ND	0.23	17.84%
		−0.53	QSQPAFLATQQSISK	15	1780–1795	HLA-DRB1*04:01, HLA-DRB1*01:01	0.61	ND	0.3	22.06%
	HMW3	0.68	TPIASRFTGVTPMAV	15	573–588	HLA-DRB1*01:01, HLA-DRB1*04:01, HLA-DRB1*07:01, HLA-DRB1*09:01	0.52	ND	0.49	43.06%
	p1	−0.48	WAPRPWAAFRGSWVN	15	1160–1175	HLA-DRB1*09:01	0.68	ND	0.98	6.40%

ND: not detected.

**Table 2:** Physicochemical properties, immunogenicity and secondary structure of the multi-epitope vaccine.

Physical and Chemical Properties	Instability and Theoretical pI	Immunoreactivity	Secondary Structure
Number of amino acids: 458	Instability index (II): 31.46	Non-allergen	α-helix: 46.07% (211/458)
Molecular weight: 50,110.47	Aliphatic index: 76.29	Immunogenicity: 5.96	β-strand: 12.23% (56/458)
Predicted scaled solubility: 0.34	Theoretical pI: 5.34	Antigen: 0.62	Random coils: 41.7% (191/458)
Grand average of hydropathicity (GRAVY): −0.22			

**Table 3:** List of residue pairs in the constructed vaccine that are capable of forming disulfide bonds, along with their energy scores and chi-3 (dihedral angles).

Res1 AA	Res2 AA	chi-3	Energy (kcal/mol)
Pro23	Asp28	90.23	1.96
Asn25	Asp28	125.88	4.21
Ile26	Ser121	126.22	3.66
Cys30	Cys107	127	5.02
Tyr33	Thr36	105.08	1.34
Glu104	Ala123	96.73	3.61
Lys105	Ser121	94.14	4.35
Cys107	Ala118	−114.76	4.28
Val108	His115	81.37	3.42

## Data Availability

The raw data in this study have been deposited in GitHub under https://github.com/xielisos567/MP_dataset, accessed on 19 February 2026. The other data supporting the findings of this study are available within the paper and additional files.
